# P04.38. The efficacy of an English-to-Danish translation of a low-dose mindfulness workplace intervention for Scandinavian bank employees on stress

**DOI:** 10.1186/1472-6882-12-S1-P308

**Published:** 2012-06-12

**Authors:** M Klatt, C Norre, S White

**Affiliations:** 1 The Ohio State University College of Medicine, Columbus, USA; 2 Copenhagen University, Copenhagen, Denmark

## Purpose

To determine the impact of a low-dose mindfulness based intervention, translated from English to Danish, delivered at the worksite on stress, sleep, and work engagement, for a group of Scandinavian bank employees.

## Methods

In a randomized, wait-list control longitudinal design, employees from a large Scandinavian bank (n=57) were recruited for a standardized worksite low-dose Mindfulness-Based Intervention (MBI). Participants, randomized and stratified to group by gender, were 39% middle-upper management employees, 41% support staff or consultant status. Mean age was 43 years with 31% males and 69% females. Changes in stress were evaluated pre/post intervention and 8 weeks post intervention via the Perceived Stress Scale (PSS), sleep quality via the Pittsburg Sleep Quality Index (PSQI), and work engagement, using Utrecht Work Engagement Scale-9 (UWES-9).

## Results

A significant group x time effect was observed for PSS scores (p<0.001) as the treatment group decreased from 19.00 (sd = 5.46) to 14.07 (sd = 4.92) after treatment while the control group showed virtually no change during that time period. There was also a significant decrease (increase in sleep quality) in the PSQI (p=0.005) for the intervention group only, as scores decreased from 5.93 (sd=1.80) to 3.89 (sd=1.60) after treatment. In sleep quality sub scales, significant shifts in the treatment group were noted in the subjective sleep quality component (p = 0.007) and daytime dysfunction (p=0.004). At 2 months after the intervention ended, no additional significant changes in the PSS, PSQI or the PSQI components were observed, but nor did scores return to pre treatment values for the intervention group.

## Conclusion

A low-dose standardized MBI translated into Danish was effective in helping Scandinavian bank employees manage stress, have better quality of sleep, and be more awake and functional during work hours. This standardized MBI was effective beyond its cultural/language origin in addressing workplace stress.

